# Sleep problems at ages 8–9 and ADHD symptoms at ages 10–11: evidence in three cohorts from INMA study

**DOI:** 10.1007/s00431-023-05145-3

**Published:** 2023-09-18

**Authors:** Llúcia González-Safont, Marisa Rebagliato, Ane Arregi, Paula Carrasco, Mònica Guxens, Oscar Vegas, Jordi Julvez, Marisa Estarlich

**Affiliations:** 1 Nursing and Chiropody Faculty of Valencia University, Nineteenth of Menéndez Pelayo St., 46010 Valencia, Spain; 2 CIBER de Epidemiología y Salud Pública (CIBERESP), Fifth of Monforte Lemos St., 28029 Madrid, Spain; 3 JRU in Epidemiology, Environment and Health FISABIO-UJI-UV, Valencia, Spain; 4 Predepartamental Unit of Medicine of Universitat Jaume I, Faculty of Health Sciences of Jaume I University, Sos Baynat Av, 12071 Castelló, Spain; 5 University of the Basque Country, UPV/EHU, 48940 Leioa, Spain; 6 Biodonostia Health Research Institute, Dr. Beguiristain Av, 20014 San Sebastian, Spain; 7 ISGlobal, Barcelona, Biomedical Research Park (PRBB), Eighty-Eighth Doctor Aiguader Av, 08003 Barcelona, Spain; 8 Universitat Pompeu Fabra, Barcelona, Spain; 9 Department of Child and Adolescent Psychiatry/Psychology, Erasmus MC, University Medical Centre, Rotterdam, The Netherlands

**Keywords:** Sleep problems, ADHD, Childhood, Preadolescence, Cohort study

## Abstract

Sleep problems and attention deficit hyperactivity disorder (ADHD) are interrelated during childhood and preadolescence. The objective of this work is assessing if sleep problems at ages 8–9 represent an alarm sign for presenting ADHD problems at ages 10–11 in three cohorts from INMA Study. Participants were 1244 children from Gipuzkoa, Sabadell, and Valencia cohorts. Sleep problems were assessed (ages 8–9) with the sleep items of the Child’s Behaviour Checklist (CBCL), and ADHD problems were collected through the Conner’s Parent Rating Scales-Revised: Short Form (CPRS-R:S) (age 10–11). Minimally and fully adjusted negative binomial models were fitted for each CPRS-R:S scale. Linearity of the relationship was assessed with generalized additive models (cubic smoothing splines with 2, 3, and 4 knots). For sensitivity analyses, children with previous symptoms, those born preterm and small for gestational age, and cases with extreme values, were excluded. Sleep problems presented IRR (95% CI) of 1.14 (1.10–1.19), 1.20 (1.14–1.26), 1.18 (1.11–1.25), and 1.18 (1.13–1.23) for opposition, inattention, hyperactivity, and ADHD scales, respectively. Fully adjusted models slightly decreased the IRR, but the association remained similar and significant. Sensitivity analyses showed similar results to fully adjusted models with only hyperactivity shown a slight decrease on significance (*p* = 0.051) when ADHD cases at age 9 were excluded.

*Conclusion*: Sleep problems are an alarm sign for later neurodevelopment problems such as ADHD. Healthcare systems could take advantage implementing policies to pay special attention on the sleep habits and sleep hygiene. This could contribute to add evidence to public health programmes such as the Healthy Child Programme.

**Supplementary Information:**

The online version contains supplementary material available at 10.1007/s00431-023-05145-3.

**Table:** 

**What is Known:** *• Sleep problems and Attention Deficit Hyperactivity Disorder (ADHD) are common problems during childhood and preadolescence, with prevalences of 20-40% and 3.0-7.5%, respectively.* *• Sleep problems are even more common in children with ADHD condition.*	
**What is New:** *• Sleep problems at ages 8-9 increased the risk of ADHD symptoms at ages 10-11.* *• This association remained strong even when considering child’s previous (5 years) and concurrent (8-9 years) ADHD symptoms, and other clinical conditions, such as being born preterm or small for gestational age.*	

## Introduction

Sleep is “a normal, reversible, recurrent state of reduced responsiveness to external stimulation that is accompanied by complex and predictable changes in physiology” [1], necessary for physical and mental performance [2] (involving in learning, declarative and procedural memory, generalization of knowledge, and emotional processing) [3]. Good sleep habits are essential during childhood, as it is a sensitive period to brain maturation and cognitive development [4]. Sleep problems are difficulties initiating, maintaining, and reinitiating sleep or returning to wakefulness [5]. They have immediate (parental stress, child’s mental health, and academic achievement) [6] and lifelong consequences (e.g. mood and behaviour problems, worsened cognitive performance, more risk of obesity) [7]. Sleep problems are common in healthy children and preadolescents [8], with prevalences of 24–40% [9, 10] and 20%, respectively [9].

In preadolescence, there are influences which may alter quantity, quality, and schedule of sleep: (A) the brain reduces synaptic density and increases white matter; (B) sleep architecture and circadian rhythms shift; and (C) poor sleep hygiene is more common (inconsistent sleep schedule, inappropriate conditions of temperature and noise, etc.) [7]. These changes can alter sleep patterns [11], being the most frequent problems: bedtime resistance, sleep onset delay, sleep-related anxiety, night waking and parasomnia in young healthy children [8, 9], and daytime sleepiness in older healthy adolescents [9].

Attention deficit hyperactivity disorder (ADHD) is a disorder characterized by symptoms of inattention (inability to concentrate), hyperactivity (excessive movement inappropriate to a situation or excessive fidgeting, tapping, or talking), and/or impulsivity (acting without thinking, in a way that may be potentially dangerous) [12], which should be present in at least two different environments (e.g. home, school, friends) for at least 6 months [13]. According to the World Health Organization (WHO) and the Diagnostic and Statistical Manual for Mental Disorders Fifth Edition (DSM-5), its prevalence is around 3–5% in childhood and 5.9–7.1 in adolescence [14] and is classified by DSM-5 in three subtypes: hyperactive-impulsive, inattentive, and combined.

Sleep problems are even more common in children with ADHD symptoms, with their prevalence increasing to 25–73.3% of children with this disorder [6, 11, 15, 16]. These children present circadian rhythm sleep–wake disorders, sleep-related breathing disorders, delayed chronotype, narcolepsy [17], and insomnia, being the most common problem in children with ADHD (73.3%) [16]. The relationship between ADHD symptoms and sleep problems is controversial, as bidirectional relationships between the two types of problems have been observed [11].

On the one hand, sleep problems could be producing ADHD-like symptoms. Sleep problems drive to sleep deprivation, which provokes a low activation of the prefrontal cortex. This leads to oxidative stress and neuronal loss and impact on cognitive functions [2, 3] (showing poorer executive functions and poorer impulse control regulation). Some studies indicate that these symptoms mimic typical ADHD symptoms [4, 11, 18].

On the other hand, children with more severe ADHD present more sleep problems [19], but also a bad sleep quality may worsen ADHD symptoms [15]. Children with ADHD may present specific circadian rhythms and chronotypes that differ from neurotypical subjects [19], and physical (obstructive sleep apnoea [7], restless legs syndrome [11], periodic limb movement [20]) [15] and psychological (opposition, anxiety, etc.) [11, 15] comorbidities may cause difficulty in sleeping. In addition, the use of metylphenidate (psychostimulant drug which improves concentration and decreases hyperactivity) [21] could difficult the sleep onset [11, 18, 22].

Objective measurements of sleep can be expensive in time and cost [17], and simple and massive assessments such as the sleep items of Child Behaviour Checklist (CBCL) could be employed, as are correlated with objective measurements [23]. There is a lack of studies examining the nature of this relationship in a Spanish framework, considering diverse geographical areas with large samples. This work will contribute to provide more evidence on sleep problems and will assess if they could be considered as an alarm sign for presenting later ADHD symptoms. It will allow the implementation of public health guidelines and the development of sleep problems screening tools as a preventive step for further neurodevelopment problems. The objective of this study is twofold: (a) to analyse whether sleep problems at 8–9 years of age are related to ADHD symptoms at 10-11 years of age in three cohorts of the INMA Study: Gipuzkoa, Sabadell, and Valencia and (b) to evaluate the influence of pre-existent clinical conditions and the potential effect modification by sociodemographic characteristics and sex on this relationship.

## Method

### Study design

**Fig. 1:**
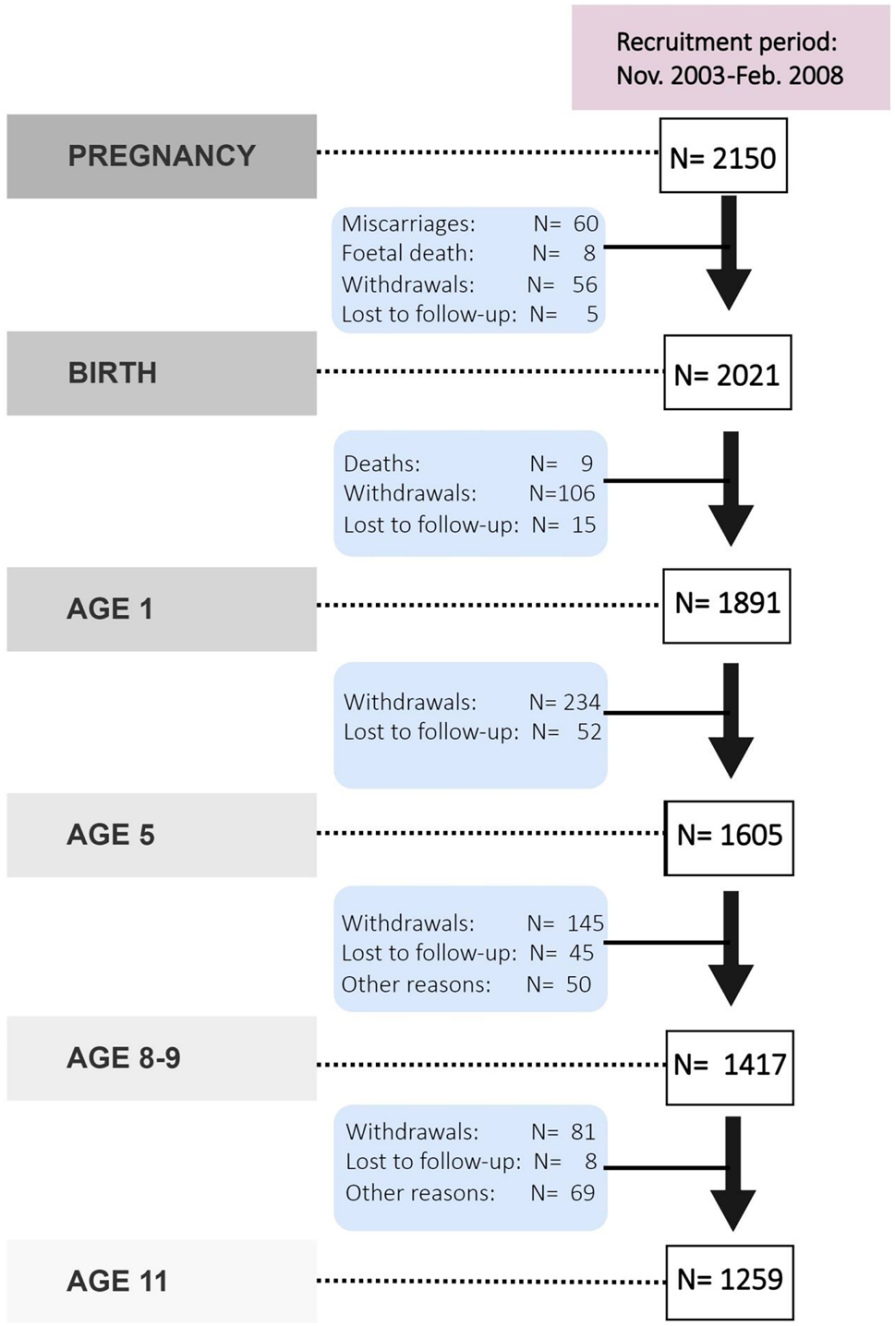
Number of participants and follow-up visits

The INMA Study is a Spanish population-based mother-and-child multicentre cohort set up in 2003 in 7 areas. This work uses data from INMA Valencia, Sabadell, and Gipuzkoa. Recruitment and subsequent procedures are described elsewhere [24]. Briefly, mothers were recruited during their first prenatal visit to their reference hospital before week 13 of gestation. The inclusion criteria were at least 16 years of age, 10–13 weeks of gestation, singleton pregnancy, intention of undergoing follow-up and delivery at the corresponding reference hospital, and no impediment for communication. Baseline participants were collected between November 2003–June 2005, June 2004–September 2006, and May 2006–February 2008, in Valencia (*N* = 855), Sabadell (*N* = 657), and Gipuzkoa (*N* = 638), respectively. Successive follow-up visits and sample evolution are described in Fig. 1 for the joint cohorts and in Supplementary Material (Fig. 1) for each cohort separately. Follow-ups were approved by local institutional ethical review boards (Centro Superior en Salud Pública, Parc de Salut Mar, and the Euskadi Clinical Research Ethics Committee, respectively), and participants gave their consent to participate. This study was carried out in line with the principles embodied in the Declaration of Helsinki.

### Sleep problems

Child Behaviour Checklist 6–18 (CBCL 6–18) [25] was used to measure sleep problems when children were 8–9 years. This questionnaire has 113 items and was responded by mothers, who rated each item as occurring 0 “Never”, 1 “Sometimes”, and 2 “Always” in the 6 previous months. The whole instrument has good psychometric properties (Chronbach’s alpha coefficient < 0.90). We employed the seven sleep items (47, nightmares; 54, overtired without good reason; 76, sleeps less than most kids; 77, sleeps more than most kids during day and/or night; 92, talks or walks in sleep; 100, trouble sleeping (describe); and 108, wets the bed) that were summed up describing the total sleep problems (range 0–9). These items have been employed previously [23, 26–29] and present good convergent validity with other sleep questionnaires, such as the sleep disorders scale for children [27]. In addition, CBCL sleep items are easy to collect, and correlate with actigraphy on sleep latency, total sleep minutes, and in polisomnography on number of arousals, total sleep time, and sleep latency [23].

### ADHD symptoms

Conner’s Parent Rating Scales-Revised: Short Form (CPRS-R:S) was employed to measure ADHD symptoms when children were 10–11 years old. It consists in 27 items rated by mothers about her child’s behaviour in the last month. Each item was responded as 0 “Not true at all”, 1 “Just a little true”, 2 “Pretty much true”, and 3 “Very much true”. Inattention, hyperactivity, opposition, and ADHD index raw scores were employed in this work [30]. These scales are the sum of 7, 6, 6, and 12 items, respectively, which consequently present scores ranging 0–21, 0–18, 0–18, and 0–36 in each case. This instrument has been validated in children and is widely employed in Spanish population [31].

### Covariates

Family and parental characteristics as well as perinatal and child characteristics were collected by means of medical records and structured questionnaires at different follow-up visits (weeks 12 and 32 of pregnancy, birth, and ages 1, 5, 8–9, and 10–11).

Parental age and country of birth, occupational social class [32], parental education, and alcohol intake and parental tobacco use were collected at pregnancy for each parent separately. Sex, age, preterm birth (< 37 gestational weeks), parity, and small for gestational age for weight (SGAw) and head circumference (SGAhc) were obtained from medical records. Duration of breastfeeding was collected at age 1. The main care provider and number of siblings and parental mental health and intelligence were assessed with the “Symptom Checklist-90 Revised” (SCL-90-R) [33]) and the similarities subtest of the Wechsler Adult Intelligence Scale (WAIS-III) [34], respectively, at child’s age 5. The employment status, parental tobacco use, family type, and building density were recalled at ages 10–11.

Previous symptoms of ADHD were assessed with the ADHD DSM-IV formlist [35] and the CPRS-R:S [30] at child’s ages 5 and 8–9, respectively. The ADHD DSM-IV formlist was responded by the child’s teacher, and clinical symptoms were obtained (answered items were transformed: 0–1 as 0 and 2–3 as 1). Children with scores > 5 for inattention (items 1–9) and hyperactivity/impulsivity (items 10–18) were classified as ADHD inattentive and hyperactive/impulsive types, respectively. Children with scores > 11 for the whole scale were classified as combined type. Children not fulfilling any of these criteria were classified as not having ADHD. In our analyses, we employed a categorization of ADHD vs non-ADHD. For each scale of the CPRS-R:S, we classified cases as being at risk (T score > 65) vs not being at risk, according to the manual [30].

### Analyses

For descriptive analyses, frequencies and percentages were used for categorical variables, while medians and interquartile ranges were used for continuous variables. For bivariate analyses, Mann-Whitney *U* and Kruskal-Wallis tests were used to assess the relation between categorical covariates and ADHD scores. To assess the bivariate relation of continuous covariates and CBCL’s sleep problems ADHD scores, Spearman correlations were employed.

We evaluated the linearity of the association between sleep problems and ADHD score, comparing linear and non-linear models (cubic smoothing splines with 2, 3, and 4 knots) with the aid of graphical examination and the AIC test. Because non-linear models did not provide a better fit, the relationship between sleep problems and ADHD symptoms was assessed linearly by a negative binomial regression (the best GAM models are represented in Supplementary material, Figs. 2–5). This provided the incidence rate ratio (IRR), which can be interpreted as the % increase or decrease in ADHD scores per one-unit change in the CBCL’s sleep problems. Cohort and child’s age and sex were included in all models regardless of their statistical significance. Several steps were undertaken in the regression models: (1) Initial models were performed considering covariates significantly related to the ADHD scores at *p* < 0.20 in the bivariate analyses and consecutively excluding those variables with a *p* value > 0.20 in the adjusted model based on the likelihood ratio test. (2) Sleep problems were included in each of the regressions of the previous step, to assess its effect on ADHD scores.

Several sensitivity analyses were performed, excluding children with previous clinical problems (cases born SGAw, SGAhc, or preterm) and children with previous symptoms of ADHD. The fully adjusted models were re-ran excluding extreme values (calculated as median ± 3*IQR) for sleep and CPRS-R:S scales. A supplementary analysis was performed repeating the sensitivity analyses but employing the hit reaction time standard error (HRT-SE) from the attention network and flanker tasks (see Supplementary Methods and Fig. 7).

Interactions were also checked in the fully adjusted models to assess the role of potential key variables (sex, cohort, paternal social class, maternal education, paternal employment, family type), and if there was any effect modifier (considered at *p* < 0.050), we performed stratified models and represented them graphically by the interaction variable. Statistical analyses were performed with IBM SPSS Statistics package version 26, R and RStudio (versions 4.1.3 and 2022.02.3 + 492, respectively) with the packages of MASS, haven, foreign, ggplot2, mgcv, and sjPlot. Flowcharts were designed with draw.io.

## Results

### Descriptive analysis

Information on child’s sleep and ADHD problems was available for 1307 and 1204 cases, respectively. Most parents were included in the secondary education category (40.6% of mothers and 43.6% of fathers) (Table 1) and belonged to the lowest social class (42.5 and 58.3 for maternal and paternal social class). The majority of parents were employed (78.5% of mothers and 90.2% of fathers) and were born in Spain. Parents usually lived together with one or more children, and nearly half of the children were females. These characteristics diverged across cohorts, with the exception of child’s sex. A regional trend was observed, with Gipuzkoa presenting a less deprived population and less migrant parents, followed by Sabadell and Valencia. Biparental families were more frequent in Gipuzkoa, and Sabadell had a higher proportion of only children. For further information on covariates, see Supplementary material (Table 1).

**Table 1:** Sociodemographic characteristics and key variables across cohorts

		**All participants**			**Gipuzkoa**			**Sabadell**			**Valencia**			
		***N***	**%**		***N***		**%**	***N***		**%**	***N***		**%**	***P* value**^**a**^
	Primary	218	19.3		32		9.28	104		23.42	82		24.12	
Maternal education	Secondary	458	40.6		128		37.10	185		41.67	145		42.65	< 0.001
	University	453	40.1		185		53.62	155		34.91	113		33.24	
	Primary	356	31.4		72		21.05	144		31.79	140		41.42	
Paternal education	Secondary	494	43.6		168		49.12	196		43.27	130		38.46	< 0.001
	University	283	25		102		29.82	113		24.94	68		20.12	
	Highest (CS I + II)	288	26.4		119		34.39	98		24.14	71		20.88	
Maternal social class	Middle (CS III)	340	31.1		104		30.06	138		33.99	98		28.82	< 0.001
	Lowest (CS IV + V)	464	42.5		123		35.55	170		41.87	171		50.29	
	Highest (CS I + II)	258	24.2		98		28.49	98		25.45	62		18.29	
Paternal social class	Middle (CS III)	187	17.5		45		13.08	72		18.70	70		20.65	0.005
	Lowest (CS IV + V)	623	58.3		201		58.43	215		55.84	207		61.06	
	Highest (CS I + II)	419	38.4		172		49.71	145		35.71	102		30.00	
Family social class	Middle (CS III)	304	27.8		77		22.25	127		31.28	100		29.41	< 0.001
	Lowest (CS IV + V)	369	33.8		97		28.03	134		33.00	138		40.59	
Maternal employment at child’s age 11	Working	894	78.5		307		88.73	365		79.52	222		66.47	< 0.001
	Not working	245	21.5		39		11.27	94		20.48	112		33.53	
Paternal employment at child’s age 11	Working	937	90.2		327		96.18	363		86.02	247		89.17	< 0.001
	Not working	102	9.8		13		3.82	59		13.98	30		10.83	
Maternal country of birth	Spain	1028	95.5		334		98.82	370		92.73	324		95.29	< 0.001
	Not Spain	49	4.5		4		1.18	29		7.27	16		4.71	
Paternal country of birth	Spain	995	93.5		328		98.80	362		91.41	305		90.77	< 0.001
	Not Spain	69	6.5		4		1.20	34		8.59	31		9.23	
	Mother + father	947	82.9		322		93.06	352		76.86	273		80.77	
Family type (11 years)	Mother + another co-living partner	51	4.5		6		1.73	31		6.77	14		4.14	< 0.001
	Mother + another non co-living partner	55	4.8		5		1.45	30		6.55	20		5.92	
	Mother only	89	7.8		13		3.76	45		9.83	31		9.17	
Number of siblings (age 5)	0	308	27.9		58		18.35	140		30.84	110		32.93	
	1	700	63.4		224		70.89	280		61.67	196		58.68	< 0.001
	2	89	8.1		30		9.49	32		7.05	27		8.08	
	3	7	0.6		4		1.27	2		0.44	1		0.30	
Sex	Female	582	50.8		183		52.89	219		47.71	180		52.94	0.226
	Male	563	49.2		163		47.11	240		52.29	160		47.06	

		**Md**^**b**^	**P25**	**P75**	**Md**^**b**^	**P25**	**P75**	**Md**^**b**^	**P25**	**P75**	**Md**^**b**^	**P25**	**P75**	***P* value**^**c**^
Child’s age		10.9	10.6	11.1	10.7	10.5	10.8	11.0	10.5	11.5	11.0	10.9	11.1	< 0.001
Maternal age at pregnancy		31.0	29.0	33.0	31.0	29.0	33.0	31.0	28.0	33.0	31.0	28.0	34.0	0.011
Paternal age at pregnancy		32.0	30.0	36.0	33.0	31.0	37.0	32.0	29.0	35.0	32.0	29.0	35.0	< 0.001
Conner’s opposition scale (age 11)		3.0	1.0	5.0	3.0	1.0	5.0	3.0	1.0	6.0	2.0	0.0	4.0	< 0.001
Conner’s inattention scale (age 11)		2.0	0.0	5.0	1.0	0.0	5.0	3.0	1.0	6.0	2.0	0.0	5.0	< 0.001
Conner’s hyperactivity scale (age 11)		1.0	0.0	3.0	1.0	0.0	3.0	1.0	0.0	4.0	0.0	0.0	3.0	< 0.001
Conner’s ADHD scale (age 11)		5.0	2.0	11.0	4.0	2.0	9.0	6.0	3.0	13.0	4.0	1.0	9.0	< 0.001
Sleep problems (CBCL) (age 9)		1.0	0.0	2.0	1.0	0.0	2.0	1.0	0.0	2.0	1.0	0.0	2.0	0.363

*ADHD*, attention deficit hyperactivity disorder

*CBCL*, Child Behaviour Checklist

^a^*P* value from chi-squared, differences between cohorts

^b^Median

^c^*P* value from Kruskal–Wallis, differences between cohorts

Child’s median and interquartile range (IQR) age was 10.9 (10.6–11.1). Parents’ ages during pregnancy were 31 (29–33) and 32 (30–36) for mothers and fathers, respectively; however, children were slightly younger, and parents were slightly older in Gipuzkoa.

Sleep problems, opposition, inattention, hyperactivity, and ADHD presented a median and IQR of 1 (0–2), 3 (1–5), 2 (0–5), 1 (0–3), and 5 (2–11), respectively. Sleep problems did not differ across cohorts. Opposition was lower in Valencia, inattention was lower in Gipuzkoa, and in Sabadell, hyperactivity was more dispersed, and ADHD was higher.

### Regression models

Minimally and fully adjusted models for ADHD problems are shown in Table 2. Minimally adjusted models examined the relation of sleep problems considering cohort, sex, and age. These models have shown IRR (CI 95%) of 1.14 (1.10–1.19) for opposition, 1.20 (1.14–1.26) for inattention, 1.18 (1.11–1.25) for hyperactivity, and 1.18 (1.13–1.23) for ADHD. After adjusting for specific covariates in the fully adjusted models, the IRR kept their magnitude and significance.

**Table 2:** Sleep problems and their relationship with Conner’s scales in unadjusted and adjusted models

	Minimally adjusted^a^				Fully adjusted^b^			
Sleep problems (CBCL) (age 9)	IRR	95% CI		*P* value^c^	IRR	95% CI		*P* value^c^
		Lower	Upper			Lower	Upper	
Conner’s opposition scale (age 11)	1.14	1.10	1.19	< 0.001	1.12	1.07	1.18	< 0.001
Conner’s inattention scale (age 11)	1.20	1.14	1.26	< 0.001	1.16	1.09	1.23	< 0.001
Conner’s hyperactivity scale (age 11)	1.18	1.11	1.25	< 0.001	1.10	1.03	1.18	< 0.001
Conner’s ADHD scale (age 11)	1.18	1.13	1.23	< 0.001	1.11	1.06	1.17	< 0.001

*CBCL* Child Behaviour Checklist

^a^Minimally adjusted models included sleep, cohort, sex, and age variables

^b^Adjusted models included unadjusted models + core models for each Conner’s scale

Opposition was further adjusted by paternal employment, age, and mental health and maternal smoking during pregnancy and mental health

Inattention was further adjusted by paternal social class, employment, age, and mental health; maternal intelligence and mental health; family structure, being SGA (weight); and weeks of breastfeeding

Hyperactivity was further adjusted by paternal employment and mental health; maternal intelligence and mental health; family structure, being SGA (weight); and weeks of breastfeeding

ADHD was further adjusted by maternal education and mental health; paternal employment, age, and mental health; and being SGA (weight)

^c^*P* value from Wald test

**Fig. 2:**
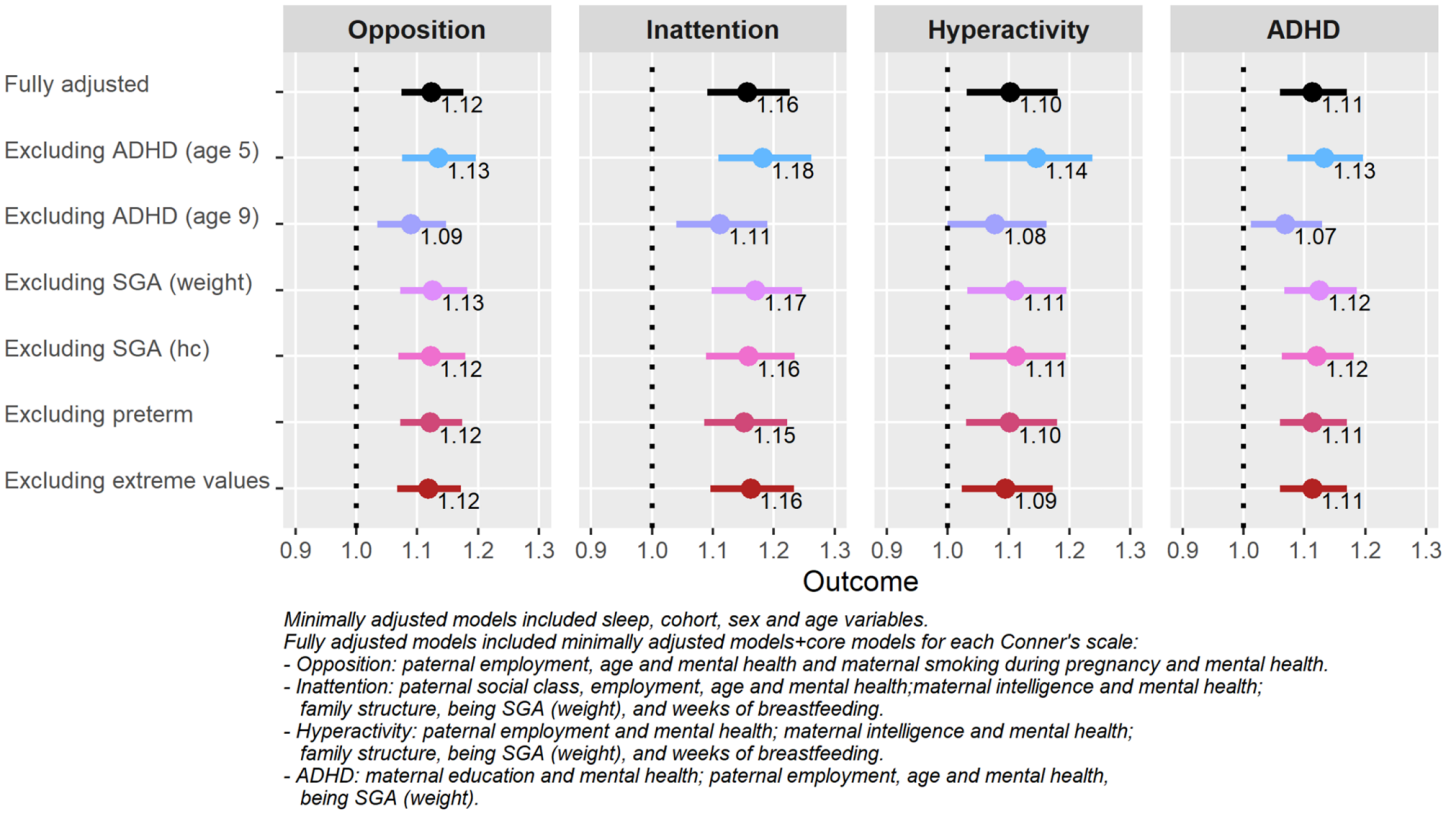
Sensitivity analyses

In the sensitivity analyses (Fig. 2, and Supplementary material, Table 2), results did not change greatly, and also kept their significance when cases detected as having previous ADHD symptoms (at 5 and 9 years of age), SGAw, SGAhc, and preterm children were excluded from the analysis. Hyperactivity model presented a slight decrease of significance (*p* value = 0.051) when ADHD cases at ages 8-9 were excluded. Analyses excluding extreme values did not change the fully adjusted models. In general terms, sleep items describing overtiredness, sleeping less or more than other kids, and trouble sleeping were those more related to Conner’s scales (see Supplementary Table 3). Supplementary analyses (see Supplementary Methods and Supplementary Fig. 6) showed a decrease in the magnitude but not in the trend or significance of the sleep problems.

**Fig. 3:**
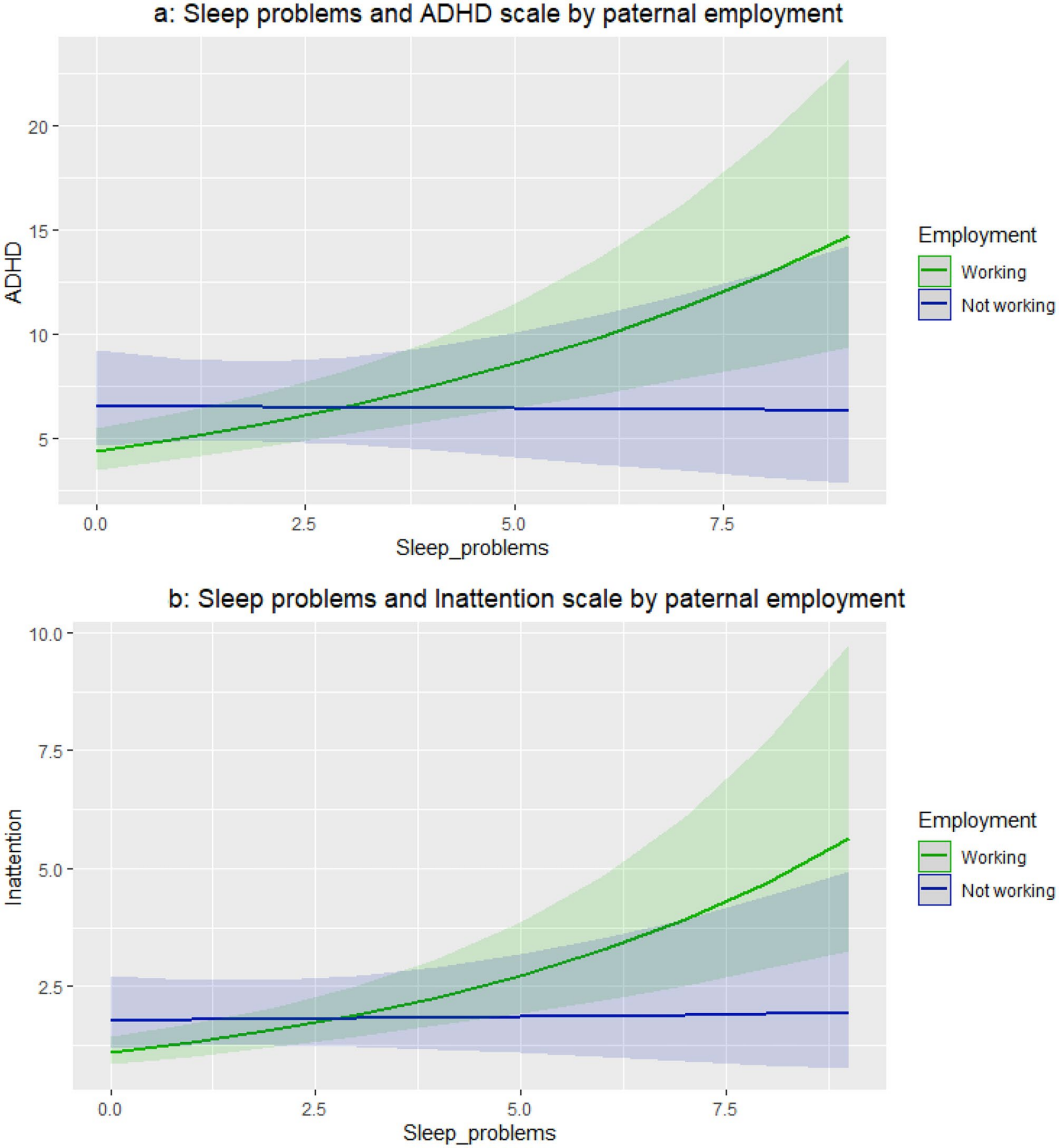
**a **Sleep problems and ADHD scale by paternal employment **b** Sleep problems and inattention scale by paternal employment

Those children from employed fathers presented a greater influence of sleep problems on ADHD problems (*p* value: 0.018) (Supplementary material, Fig. 7). Similar results can be observed for inattention scale (interaction *p* value = 0.011). We stratified and represented our ADHD and inattention models by paternal employment (see Fig. 3). Those children from non-working fathers presented higher scores of ADHD and inattention in the absence of sleep problems, in comparison to children from working fathers. With the increase of sleep problems, ADHD and inattention scores of children from working fathers tend to increase, while those from non-working fathers remained stable.

## Discussion

In this work, we explored the relation of sleep problems during childhood and the relationship on later ADHD problems, considering all opposition, inattention, hyperactivity, and overall ADHD. We developed covariate models for each outcome, and explored the potential effect of clinical conditions, finding very small changes in comparison to fully adjusted models. We also checked for potential interactions within the models, and we found that children from employed fathers presented a positive relationship between sleep problems and inattention and ADHD.

The relation between sleep problems and ADHD-like symptoms should be considered in the light of a child development perspective. Late childhood and preadolescence coincides with a development stage when intense periods of synaptogenesis and synaptic pruning [36] happen. These changes could be altering sleep slow waves [37]. It has been observed that sleep and ADHD problems have been related in this age period; however, two different perspectives are given to explain this relationship.

One perspective posits that ADHD children, due to their condition, present alterations in their sleep function [38], and the second of these perspectives states that sleep problems during childhood could be a sign alarm for later ADHD problems [21, 39]. Alteration in circadian rhythms could be more common in these ages, and therefore, the social constraints (forcing children and pre-adolescents to keep a specific schedule, for example) could induce sleep deprivation [2, 4, 16] and provoke more oxidative stress. Oxidative stress could be, in turn, affecting attentional resources and emotional liability and then producing ADHD-like symptoms [21].

We observed a relation between sleep and ADHD problems and tried to disentangle it by collecting information on sleep at ages 8–9 and ADHD problems at ages 10–11. To provide more robustness to our findings, we performed sensitivity analyses excluding cases with some clinical problems or pre-existent symptoms, and also those with extreme values in both sleep and ADHD. Results of these analyses were similar to fully adjusted models, inviting us to consider that in our sample, sleep problems could be considered as an alarm sign presented previously to future ADHD problems. We also repeated the analysis assessing inattentiveness, and the effect of sleep problems was also observed.

Regarding measurements performed by questionnaire, several works pointed in the same direction that our findings. When assessing these problems in young children, two recent works found that consistent short sleep duration was related with later ADHD symptoms in children below 6 years of age [40, 41]. However, the differences between ages could be making comparisons more difficult. Firstly, because different age ranges may present diverse psychological traits. Secondly, because ADHD should not be diagnosed at very young ages, early ADHD diagnoses could be rendering false-positive cases, especially when they are considered before age 6. Children below this ages change their behaviour rapidly as they are in constant learning of social norms. In addition, behaviours described in this syndrome such as hyperactivity, impulsivity, or inattention may be quite common in infancy and could evolve naturally as children grow up. When considering these problems in adolescents, there are two cross-sectional studies: a recent paper which found that parent-reported sleep problems were related to more ADHD severity, sluggish cognitive tempo, and irritability [5], and a research undertaken in Norway, which found more possibilities of being classified as having ADHD in late adolescence if sleep problems had been reported [42].

Three works resulted more comparable to ours. One of them also measured sleep problems previous to ADHD, but in adolescents. It showed that sleep problems (short sleep duration, insomnia, poor sleep quality, snoring) were related to ADHD symptoms even after adjusting for other covariates [43]. The second work considered the trajectory of sleep problems in 1400 Australian children, finding that ADHD problems were more common at age 10 in those children with persistent sleep problems [10]. A third work developed in the Netherlands, which collected sleep problems at age 9 through the CBCL, related its items individually to attention problems. This scale presented higher scores in those who slept less and had trouble sleeping, like in our case. They also found that nightmares were related, while we did not find this association. In the Dutch research, when analyses were adjusted by other covariates, these trends disappeared, being significant only those related to talking and walking in sleep. We did not find this trend, maybe due to the few cases which presented always this problem [28]. To our knowledge, no other studies explored this relationship through questionnaire in two stages during middle childhood and pre-puberty, considering pre-existent ADHD symptoms.

Social determinants were included in our work, and we also explored if they provoked an interaction in our models. We observed that paternal employment was an effect modifier, meaning that the effect of sleep problems on ADHD was different depending on the father’s employment situation. We further explored the linear association stratifying by father’s employment situation finding that children from working fathers presented higher ADHD risk at higher sleep problems, while children from not working fathers did not present an association between sleep problems and ADHD. One possible explanation for this is that children from non-working parents could present higher ADHD scores but the same distribution for sleep problems scores for both working and not working fathers. Presenting more ADHD symptoms in more economically disadvantaged contexts has been explored previously in the literature and coincides with the current evidence [44]. A recent work undertaken in Germany which employed a composite measure for socioeconomic status (SES) could shed some light on our findings, as it seems that SES-related sleep problems during childhood were sleep related-anxiety, parasomnia, and sleep-disordered breathing, but not the total score of sleep problems [9]. For adolescence, SES was inversely related to more bedtime difficulties, sleep behaviour difficulties, and total of sleep difficulties [9]. Our sleep problems assessment was carried out in childhood rather than in adolescence, and we did not consider sleep-related anxiety or breathing problems, so this could be a possible explanation for our findings. In addition, another possible explanation is that employment situation (recalled at child’s ages 10–11) could be a more recent source of economic stress, in comparison to social class (obtained at pregnancy).

The present work has some limitations: Firstly, we could not include some relevant factors which could be determining the relation between sleep problems and ADHD, such as parenting stress [6]. Parenting stress might be a key variable to obtain a better understanding, because it might be influencing the pattern of response in the questionnaires. But in our data, it was available only for a subgroup of families, and assessed in a different timing for each cohort, so no comparisons were possible in a pull analysis. Still, we performed an additional analysis with each cohort separately, and the effect of sleep problems on ADHD symptoms did not change significatively. Secondly, cohort studies are limited by their lost to follow up, so risks could not be generalizable in general population if representativeness is lost, as in previous works we have observed that lost to follow-up is more frequent in more economically disadvantaged families [45] We also observed that participants with missing data in the minimally adjusted models where more frequently from a foreign country of origin, non-nuclear families, and children from mothers with less score in intelligence tests. This could be biasing the effect of sleep problems in ADHD symptoms. Thirdly, we did not employ ADHD diagnoses, but as the sample was non-clinical, we considered ADHD scores as a continuous in order to avoid false-positive and to increase the power of the analyses. Finally, we did not employ objective measurements, but the CBCL sleep items have been employed in previous works [23, 26–29, 46], correlate well with objective data, and are more cost-effective [23]. Moreover, they have good convergent validity with other sleep questionnaires [27]. They have been explored in multiple combinations in previous works that consider 5 [46], 6 [23, 28, 46], or 7 [26, 27] of the sleep items depending on the child’s age and item availability (for children below 6 years of age, it is employed the CBCL 1.5–5 [47] that has less sleep items than CBCL 6–18).

The present work also presents several strengths: it includes sample from different areas in Spain, and this may avoid regional variability and could improve the power of the analyses. A whole range of factors of children’s environment that could be affecting ADHD symptoms was also included. This was possible due to its prospective nature, which allows the inclusion of information collected at different time point. One of the major strengths of this work is its specific design, employing the information on sleep problems collected in a previous time point to ADHD symptoms. This data collection in two steps vanishes the cross-sectional correlation, and the sensitivity analyses provide more strength to our findings. Lastly, the search of interactions and stratification by socioeconomic factors allowed us to observe specific patterns of the relationship studied.

The present work contributes to the evidence that sleep problems in childhood may have an influence on ADHD symptoms even at early pre-pubertal stages. However, our evidence is not strong to establish a causal mechanism, but sleep problems could be considered an alarm sign for general health, and specifically, for ADHD symptoms. Healthcare systems could take advantage of these findings, implementing policies to pay special attention on the sleep habits and sleep hygiene. This could contribute to add evidence to public health programmes such as the Healthy Child Programme. Moreover, our work employed parent-reported questionnaires to detect both sleep and ADHD problems, and this system of detection could be easily and cost-effective translated into clinical practice. If results were confirmed in a wider sample, an assessment on sleep quality could be implemented in middle childhood. Given the high prevalence of these problems in this age period, the development and modification of sleep routines could easily ameliorate sleep problems and, also, future ADHD symptoms during pre-puberty.

### Supplementary Information

Below is the link to the electronic supplementary material.

Supplementary file1 (DOCX 12249 KB)

## Data Availability

Data availability will be possible only after request to the authors and external collaboration protocol fulfillment requested by the INMA Study Executive Committee.
