# Supplementary material for: Sleep problems at ages 8–9 and ADHD symptoms at ages 10–11: evidence in three cohorts from INMA study
Source: Eur J Pediatr. 2023 Sep 18;182(11):5211–22. doi: 10.1007/s00431-023-05145-3 (PMC10640481; doi:10.1007/s00431-023-05145-3)

## Supplementary methods

As the same test was not available for all the cohorts, two comparable tests were employed in terms of cognitive function, the Attention Network Test (ANT), and the Flanker test. At 10-11 years of child’s age, ANT was applied in Valencia and Gipuzkoa, and Flanker was applied in Sabadell.

The only difference between ANT and Flanker test is the visual stimuli provided. In ANT are yellow fish, and in Flanker, black arrows. They are computerized tests that take approximately 20 minutes to complete. A row of five visual stimuli (yellow fish or black arrows) appear either above or below a fixation point are presented. Participants must press the right or the left button on the mouse depending on the direction in which the stimulus in the middle is pointing and ignoring flanker stimuli, which may point in either the same (congruent) or opposite (incongruent) direction as to the middle stimulus[1]. The target is preceded by visual signals that inform either about the upcoming of the target only (alerting cue) or about the upcoming of the target as well as its location (orienting cue)[2].

These tests provide a summary outcome, the hit reaction time standard error (HRT-SE). This outcome is a measure of speed consistency throughout the test, and higher scores indicate highly variable reactions related to inattentiveness. As part of a supplementary analysis, the sensitivity analyses were repeated employing as outcome the HRT-SE **(see supplementary figure 6)** [3].

## References from supplementary methods

1. Arora S, Lawrence MA, Klein RM (2020) The Attention Network Test Database: ADHD and Cross-Cultural Applications. Front Psychol 11:

2. Antón E, Duñabeitia JA, Estévez A, et al (2014) Is there a bilingual advantage in the ANT task? Evidence from children. Front Psychol 5:398. https://doi.org/10.3389/fpsyg.2014.00398

3. Julvez J, Fernández-Barrés S, Gignac F, et al (2020) Maternal seafood consumption during pregnancy and child attention outcomes: a cohort study with gene effect modification by PUFA-related genes. Int J Epidemiol 49:559–571. https://doi.org/10.1093/ije/dyz197

## Supplementary tables

| Supplementary table 1.Comparison between included and non-included in the minimally adjusted models | | | | | | | | | |
| --- | --- | --- | --- | --- | --- | --- | --- | --- | --- |
|  |  | | **Included** | | | **Non-included** | | |  |
|  | | | **N** | | **%** | **N** | | **%** | **P-value^a^** |
| **SOCIODEMOGRAPHIC AND FAMILY** | | | | | | | | | |
| Maternal education | | Primary | 218 | | 19.3 | 27 | | 23.9 | 0.200 |
|  |  | Secondary | 458 | | 40.6 | 50 | | 44.2 |  |
|  |  | University | 453 | | 40.1 | 36 | | 31.9 |  |
| Paternal education | | Primary | 356 | | 31.4 | 40 | | 34.5 | 0.742 |
|  |  | Secondary | 494 | | 43.6 | 50 | | 43.1 |  |
|  |  | University | 283 | | 25.0 | 26 | | 22.4 |  |
| Maternal social class | | Highest (CS I+II) | 288 | | 26.4 | 28 | | 25.7 | 0.520 |
|  |  | Middle (CS III) | 340 | | 31.1 | 29 | | 26.6 |  |
|  |  | Lowest (CS IV+V) | 464 | | 42.5 | 52 | | 47.7 |  |
| Paternal social class | | Highest (CS I+II) | 258 | | 24.2 | 21 | | 20.0 | 0.584 |
|  |  | Middle (CS III) | 187 | | 17.5 | 21 | | 20.0 |  |
|  |  | Lowest (CS IV+V) | 623 | | 58.3 | 63 | | 60.0 |  |
| Family social class | | Highest (CS I+II) | 419 | | 38.4 | 38 | | 34.9 | 0.285 |
|  |  | Middle (CS III) | 304 | | 27.8 | 26 | | 23.9 |  |
|  |  | Lowest (CS IV+V) | 369 | | 33.8 | 45 | | 41.3 |  |
| Maternal employment at child's age 11 | | Working | 894 | | 78.5 | 87 | | 82.9 | 0.294 |
|  |  | Not working | 245 | | 21.5 | 18 | | 17.1 |  |
| Paternal employment at child's age 11 | | Working | 937 | | 90.2 | 81 | | 89.0 | 0.720 |
|  |  | Not working | 102 | | 9.8 | 10 | | 11.0 |  |
| Maternal country of birth | | Spain | 1028 | | 95.5 | 98 | | 90.7 | 0.032 |
|  |  | Not Spain | 49 | | 4.5 | 10 | | 9.3 |  |
| Paternal country of birth | | Spain | 995 | | 93.5 | 86 | | 85.1 | 0.002 |
|  |  | Not Spain | 69 | | 6.5 | 15 | | 14.9 |  |
| Family type (11 years) | | Mother+father | 947 | | 82.9 | 79 | | 74.5 | 0.003 |
|  |  | Mother+ anotherco-living partner | 51 | | 4.5 | 12 | | 11.3 |  |
|  |  | Mother+ another non co-living partner | 55 | | 4.8 | 2 | | 1.9 |  |
|  |  | Mother only | 89 | | 7.8 | 13 | | 12.3 |  |
| Number of siblings (age 5) | | 0 | 308 | | 27.9 | 23 | | 23.7 | 0.002 |
|  |  | 1 | 700 | | 63.4 | 55 | | 56.7 |  |
|  |  | 2 | 89 | | 8.1 | 16 | | 16.5 |  |
|  |  | 3 | 7 | | 0.6 | 3 | | 3.1 |  |
| Sex | | Female | 582 | | 50.8 | 63 | | 53.8 | 0.534 |
|  |  | Male | 563 | | 49.2 | 54 | | 46.2 |  |
| Main care provider (age 4-5) | | Mother | 596 | | 54.2 | 56 | | 56.0 | 0.792 |
|  |  | Father | 50 | | 4.5 | 4 | | 4.0 |  |
|  |  | Both | 306 | | 27.8 | 27 | | 27.0 |  |
|  |  | Grandparents | 145 | | 13.2 | 12 | | 12.0 |  |
|  |  | Other situations | 3 | | 0.3 | 1 | | 1.0 |  |
| **TOXICANTS** | | | | | | | | | |
| Smoking during pregnancy (mother) | | No | 968 | | 85.9 | 100 | | 86.2 | 0.926 |
|  |  | Yes | 159 | | 14.1 | 16 | | 13.8 |  |
| Smoking during pregnancy (father) | | No | 721 | | 64.5 | 70 | | 62.5 | 0.666 |
|  |  | Yes | 396 | | 35.5 | 42 | | 37.5 |  |
| Smoking at child's age 11 (mother) | | No | 850 | | 74.7 | 79 | | 73.8 | 0.845 |
|  |  | Yes | 288 | | 25.3 | 28 | | 26.2 |  |
| Smoking at child's age 11 (father) | | No | 804 | | 73.8 | 78 | | 75.0 | 0.795 |
|  |  | Yes | 285 | | 26.2 | 26 | | 25.0 |  |
| Alcohol during pregnancy (mother) | | No | 977 | | 91.1 | 95 | | 88.0 | 0.275 |
|  |  | Yes | 95 | | 8.9 | 13 | | 12.0 |  |
| Alcohol during pregnancy (father) | | No | 213 | | 19.8 | 30 | | 27.8 | 0.050 |
|  |  | Yes | 863 | | 80.2 | 78 | | 72.2 |  |
| Supplementary table 1 (continued) .Comparison between included and non-included in the minimally adjusted models | | | | | | | | | |
|  |  | | **Included** | | | **Non-included** | | |  |
|  | | | **N** | | **%** | **N** | | **%** | **P-value^a^** |
| **CLINIC** | | | | | | | | | |
| Preterm | | No | 1099 | | 96.7 | 113 | | 97.4 | 0.663 |
|  |  | Yes | 38 | | 3.3 | 3 | | 2.6 |  |
| Small for gestational age (weight) | | No | 974 | | 90.3 | 101 | | 93.5 | 0.271 |
|  |  | Yes | 105 | | 9.7 | 7 | | 6.5 |  |
| Small for gestational age (head circumference) | | No | 951 | | 90.7 | 95 | | 89.6 | 0.706 |
|  |  | Yes | 97 | | 9.3 | 11 | | 10.4 |  |
| Types of ADHD (age 5) | | No ADHD | 875 | | 94.7 | 77 | | 98.7 | 0.400 |
|  |  | Inattentive | 23 | | 2.5 | 0 | | 0.0 |  |
|  |  | Hyperactive | 17 | | 1.8 | 1 | | 1.3 |  |
|  |  | Combined | 9 | | 1.0 | 0 | | 0.0 |  |
| History of ADHD (age 5) | | No | 875 | | 94.7 | 77 | | 98.7 | 0.117 |
|  |  | Yes | 49 | | 5.3 | 1 | | 1.3 |  |
| History of ADHD (age 9) | | No | 1043 | | 91.7 | 19 | | 90.5 | 0.836 |
|  |  | Yes | 94 | | 8.3 | 2 | | 9.5 |  |
|  | | | **Md^b^** | **P25** | **P75** | **Md^b^** | **P25** | **P75** | **P-value^c^** |
| **KEY VARIABLES** | | | | | | | | | |
| Child's age | | | 10.9 | 10.6 | 11.1 | 11.0 | 10.7 | 11.2 | 0.086 |
| Maternal age at pregnancy | | | 31.0 | 29.0 | 33.0 | 31.0 | 29.0 | 34.0 | 0.320 |
| Paternal age at pregnancy | | | 32.0 | 30.0 | 36.0 | 32.0 | 29.0 | 35.0 | 0.170 |
| Conner's Opposition scale (age 11) | | | 3.0 | 1.0 | 5.0 | 2.0 | 0.0 | 4.0 | 0.028 |
| Conner's Inattention scale (age 11) | | | 2.0 | 0.0 | 5.0 | 2.0 | 0.0 | 6.0 | 0.883 |
| Conner's Hyperactivity scale (age 11) | | | 1.0 | 0.0 | 3.0 | 1.0 | 0.0 | 4.0 | 0.297 |
| Conner's ADHD scale (age 11) | | | 5.0 | 2.0 | 11.0 | 5.0 | 2.0 | 11.0 | 0.426 |
| Sleep problems (CBCL) (age 9) | | | 1.0 | 0.0 | 2.0 | 0.5 | 0.0 | 1.0 | 0.341 |
| **URBAN** | | | | | | | | | |
| Urban density buffer 100 (age 11) m^2^/km^2^ (x10^3^) | | | 421.0 | 298.6 | 507.5 | 400.7 | 277.3 | 502.7 | 0.301 |
| Urban density buffer 300 (age 11) m^2^/km^2^ (x10^3^) | | | 358.3 | 232.3 | 451.3 | 306.4 | 211.1 | 447.8 | 0.109 |
| Population density (x10^3^) | | | 6.2 | 4.0 | 12.8 | 5.7 | 2.3 | 12.7 | 0.092 |
| **PARENT'S PSYCHOLOGICAL TRAITS** | | | | | | | | | |
| Maternal intelligence | | | 10.5 | 8.3 | 12.7 | 9.0 | 7.6 | 12.0 | 0.015 |
| Paternal intelligence | | | 10.9 | 8.5 | 12.7 | 8.8 | 7.9 | 10.9 | 0.081 |
| Maternal mental health | | | 0.6 | 0.4 | 1.0 | 0.7 | 0.4 | 1.0 | 0.802 |
| Paternal mental health | | | 0.5 | 0.3 | 0.8 | 0.4 | 0.2 | 0.6 | 0.044 |
| **BREASTFEEDING** | | | | | | | | | |
| Weeks of breastfeeding | | | 25.9 | 12.7 | 43.4 | 21.9 | 13.1 | 34.9 | 0.193 |
| ^a^: P-value from chi-squared, differences between cohorts. | | | | | | | | | |
| ^b^: Median | | | | | | | | | |
| ^c^: P-value from Kruskal-Wallis, differences between cohorts. | | |  |  |  |  |  |  |  |
| ADHD: Attention Deficit Hyperactivity Disorder | | | | | | | | | |
| CBCL: Child Behavior Checklist | | | | | | | | | |

| Supplementary Table 2: Sleep problems and their relationship with Conner's Scales in sensitivity analyses | | | | | | | | | | | | | | | | |
| --- | --- | --- | --- | --- | --- | --- | --- | --- | --- | --- | --- | --- | --- | --- | --- | --- |
|  | **Opposition** | | | | **Inattention** | | | | **Hyperactivity** | | | | **ADHD** | | | |
|  |  | **95%CI** | |  |  | **95%CI** | |  |  | **95%CI** | |  |  | **95%CI** | |  |
| **Model** | **IRR** | **Lower** | **Upper** | **p-value^a^** | **IRR** | **Lower** | **Upper** | **p-value^a^** | **IRR** | **Lower** | **Upper** | **p-value^a^** | **IRR** | **Lower** | **Upper** | **p-value^a^** |
| Fully adjusted | 1.12 | 1.07 | 1.18 | <0.001 | 1.16 | 1.09 | 1.23 | <0.001 | 1.1 | 1.03 | 1.18 | 0.004 | 1.11 | 1.06 | 1.17 | <0.001 |
| Excluding ADHD (age 5) | 1.13 | 1.08 | 1.2 | <0.001 | 1.18 | 1.11 | 1.26 | <0.001 | 1.14 | 1.06 | 1.24 | 0.001 | 1.13 | 1.07 | 1.2 | <0.001 |
| Excluding ADHD (age 9) | 1.09 | 1.03 | 1.15 | <0.001 | 1.11 | 1.04 | 1.19 | 0.001 | 1.08 | 1.00 | 1.16 | 0.051 | 1.07 | 1.01 | 1.13 | 0.017 |
| Excluding SGA (weight) | 1.13 | 1.07 | 1.18 | <0.001 | 1.17 | 1.1 | 1.25 | <0.001 | 1.11 | 1.03 | 1.2 | 0.005 | 1.12 | 1.07 | 1.19 | <0.001 |
| Excluding SGA (hc) | 1.12 | 1.07 | 1.18 | <0.001 | 1.16 | 1.09 | 1.23 | <0.001 | 1.11 | 1.04 | 1.19 | 0.003 | 1.12 | 1.06 | 1.18 | <0.001 |
| Excluding preterm | 1.12 | 1.07 | 1.17 | <0.001 | 1.15 | 1.09 | 1.22 | <0.001 | 1.1 | 1.03 | 1.18 | 0.005 | 1.11 | 1.06 | 1.17 | <0.001 |
| Excluding extreme values | 1.12 | 1.07 | 1.17 | <0.001 | 1.16 | 1.1 | 1.23 | <0.001 | 1.09 | 1.02 | 1.17 | 0.008 | 1.11 | 1.06 | 1.17 | <0.001 |
| ^a^: P-value from Wald’s test | | | | | | | | | | | | | | | | |

| Supplementary Table 3: Sleep items and their relationship with Conner's Scales | | | | | | | | | | | | | | | | | | | | | | | |
| --- | --- | --- | --- | --- | --- | --- | --- | --- | --- | --- | --- | --- | --- | --- | --- | --- | --- | --- | --- | --- | --- | --- | --- |
| **Model** | | **N** | **%** | **Opposition** | | | | | **Inattention** | | | | | **Hyperactivity** | | | | | **ADHD** | | | | |
|  |  |  |  |  | **95% CI** | | **p-value^a^** | **Global p-value^b^** |  | **95% CI** | | **p-value^a^** | **Global p-value^b^** |  | **95% CI** | | **p-value^a^** | **Global p-value^b^** |  | **95% CI** | | **p-value^a^** | **Global p-value^b^** |
|  |  |  |  | **IRR** | **Lower** | **Upper** |  |  | **IRR** | **Lower** | **Upper** |  |  | **IRR** | **Lower** | **Upper** |  |  | **IRR** | **Lower** | **Upper** |  |  |
| Fully adjusted | | 1145 | 100.00 | 1.12 | 1.07 | 1.18 | <0.001 | <0.001 | 1.16 | 1.09 | 1.23 | 0.000 | 0.000 | 1.10 | 1.03 | 1.18 | 0.004 | 0.004 | 1.11 | 1.06 | 1.17 | <0.001 | <0.001 |
| i47. Nightmares^c^ | Never | 722 | 63.06 | ref | ref | ref | ref | 0.211 | ref | ref | ref | ref | 0.054 | ref | ref | ref | ref | 0.490 | ref | ref | ref | ref | 0.352 |
|  | Sometimes | 399 | 34.85 | 1.10 | 0.95 | 1.26 | 0.195 |  | 1.24 | 1.04 | 1.48 | 0.016 |  | 1.09 | 0.89 | 1.33 | 0.428 |  | 1.10 | 0.96 | 1.28 | 0.182 |  |
|  | Always | 24 | 2.10 | 1.36 | 0.88 | 2.19 | 0.177 |  | 1.20 | 0.69 | 2.20 | 0.536 |  | 1.37 | 0.75 | 2.67 | 0.330 |  | 1.19 | 0.76 | 1.96 | 0.474 |  |
| i54. Overtired^d^ | Never | 1010 | 88.21 | ref | ref | ref | ref | 0.001 | ref | ref | ref | ref | 0.001 | ref | ref | ref | ref | 0.087 | ref | ref | ref | ref | 0.003 |
|  | Sometimes | 117 | 10.22 | 1.31 | 1.05 | 1.63 | 0.017 |  | 1.10 | 0.84 | 1.46 | 0.498 |  | 0.69 | 0.49 | 0.98 | 0.033 |  | 1.04 | 0.83 | 1.32 | 0.718 |  |
|  | Always | 18 | 1.57 | 1.90 | 1.23 | 3.06 | 0.005 |  | 2.67 | 1.57 | 4.88 | 0.001 |  | 1.23 | 0.65 | 2.50 | 0.545 |  | 2.14 | 1.35 | 3.59 | 0.002 |  |
| i76. Sleeps less^e^ | Never | 971 | 84.80 | ref | ref | ref | ref | 0.005 | ref | ref | ref | ref | 0.006 | ref | ref | ref | ref | 0.021 | ref | ref | ref | ref | 0.048 |
|  | Sometimes | 128 | 11.18 | 1.22 | 1.00 | 1.51 | 0.054 |  | 1.25 | 0.97 | 1.63 | 0.085 |  | 1.20 | 0.89 | 1.63 | 0.240 |  | 1.15 | 0.93 | 1.43 | 0.207 |  |
|  | Always | 46 | 4.02 | 1.55 | 1.14 | 2.13 | 0.006 |  | 1.69 | 1.17 | 2.51 | 0.007 |  | 1.75 | 1.15 | 2.76 | 0.012 |  | 1.44 | 1.04 | 2.04 | 0.032 |  |
| i77. Sleeps more^f^ | Never | 1072 | 93.62 | ref | ref | ref | ref | 0.088 | ref | ref | ref | ref | 0.016 | ref | ref | ref | ref | 0.203 | ref | ref | ref | ref | 0.044 |
|  | Sometimes | 58 | 5.07 | 1.30 | 0.97 | 1.75 | 0.086 |  | 1.24 | 0.85 | 1.86 | 0.269 |  | 1.43 | 0.93 | 2.27 | 0.105 |  | 1.31 | 0.97 | 1.80 | 0.085 |  |
|  | Always | 15 | 1.31 | 1.47 | 0.86 | 2.64 | 0.174 |  | 2.37 | 1.24 | 5.06 | 0.014 |  | 1.39 | 0.67 | 3.25 | 0.405 |  | 1.70 | 0.96 | 3.29 | 0.088 |  |
| i92. Talks or walks in sleep^g^ | Never | 995 | 86.90 | ref | ref | ref | ref | 0.241 | ref | ref | ref | ref | 0.957 | ref | ref | ref | ref | 0.904 | ref | ref | ref | ref | 0.833 |
|  | Sometimes | 137 | 11.97 | 1.03 | 0.83 | 1.26 | 0.810 |  | 1.00 | 0.78 | 1.29 | 0.984 |  | 0.99 | 0.74 | 1.33 | 0.946 |  | 1.03 | 0.84 | 1.27 | 0.796 |  |
|  | Always | 13 | 1.14 | 1.59 | 0.93 | 2.85 | 0.103 |  | 1.11 | 0.58 | 2.35 | 0.770 |  | 0.82 | 0.35 | 2.05 | 0.648 |  | 1.18 | 0.68 | 2.23 | 0.583 |  |
| i100. Trouble sleeping^h^ | Never | 1027 | 89.69 | ref | ref | ref | ref | 0.030 | ref | ref | ref | ref | 0.005 | ref | ref | ref | ref | 0.012 | ref | ref | ref | ref | 0.005 |
|  | Sometimes | 91 | 7.95 | 1.24 | 0.98 | 1.58 | 0.075 |  | 1.33 | 1.00 | 1.80 | 0.053 |  | 1.50 | 1.07 | 2.14 | 0.020 |  | 1.27 | 0.99 | 1.63 | 0.060 |  |
|  | Always | 27 | 2.36 | 1.49 | 1.02 | 2.23 | 0.041 |  | 1.84 | 1.18 | 3.01 | 0.010 |  | 1.70 | 1.00 | 3.02 | 0.058 |  | 1.70 | 1.16 | 2.59 | 0.010 |  |
| i108. Wets the bed^i^ | Never | 1043 | 91.09 | ref | ref | ref | ref | 0.062 | ref | ref | ref | ref | 0.452 | ref | ref | ref | ref | 0.055 | ref | ref | ref | ref | 0.181 |
|  | Sometimes | 72 | 6.29 | 1.15 | 0.87 | 1.52 | 0.333 |  | 0.95 | 0.68 | 1.36 | 0.781 |  | 0.81 | 0.52 | 1.27 | 0.341 |  | 0.93 | 0.70 | 1.26 | 0.632 |  |
|  | Always | 30 | 2.62 | 1.50 | 1.04 | 2.21 | 0.031 |  | 1.33 | 0.84 | 2.18 | 0.236 |  | 1.75 | 1.05 | 3.05 | 0.036 |  | 1.40 | 0.96 | 2.11 | 0.091 |  |
| ^a^: P-value from chi-squared test for each category  ^b^: P-value from likelihood ratio test for the whole item  ^c^: 47. Nightmares  ^d^: 54. Overtired without good reason  ^e^: 76. Sleeps less than most kids  ^f^: 77. Sleeps more than most kids during day and/or night  ^g^: 92. Talks or walks in sleep  ^h^: 100. Trouble sleeping  ^i^: 108. Wets the bed | | | | | | | | | | | | | | | | | | | | | | | |

## Supplementary figure captions:

**Supplementary figure 1**. Number of participants in the follow-up visits separated by cohorts.

**Supplementary figure 2**. Shape of the relation between Sleep and Inattention.

**Supplementary figure 3**. Shape of the relation between Sleep and Hyperactivity.

**Supplementary figure 4**. Shape of the relation between Sleep and Opposition.

**Supplementary figure 5**. Shape of the relation between Sleep and ADHD.

**Supplementary figure 6**. Sensitivity analyses with ANT/Flanker.

**Supplementary figure 7**. Final models stratified.

## Supplementary figures


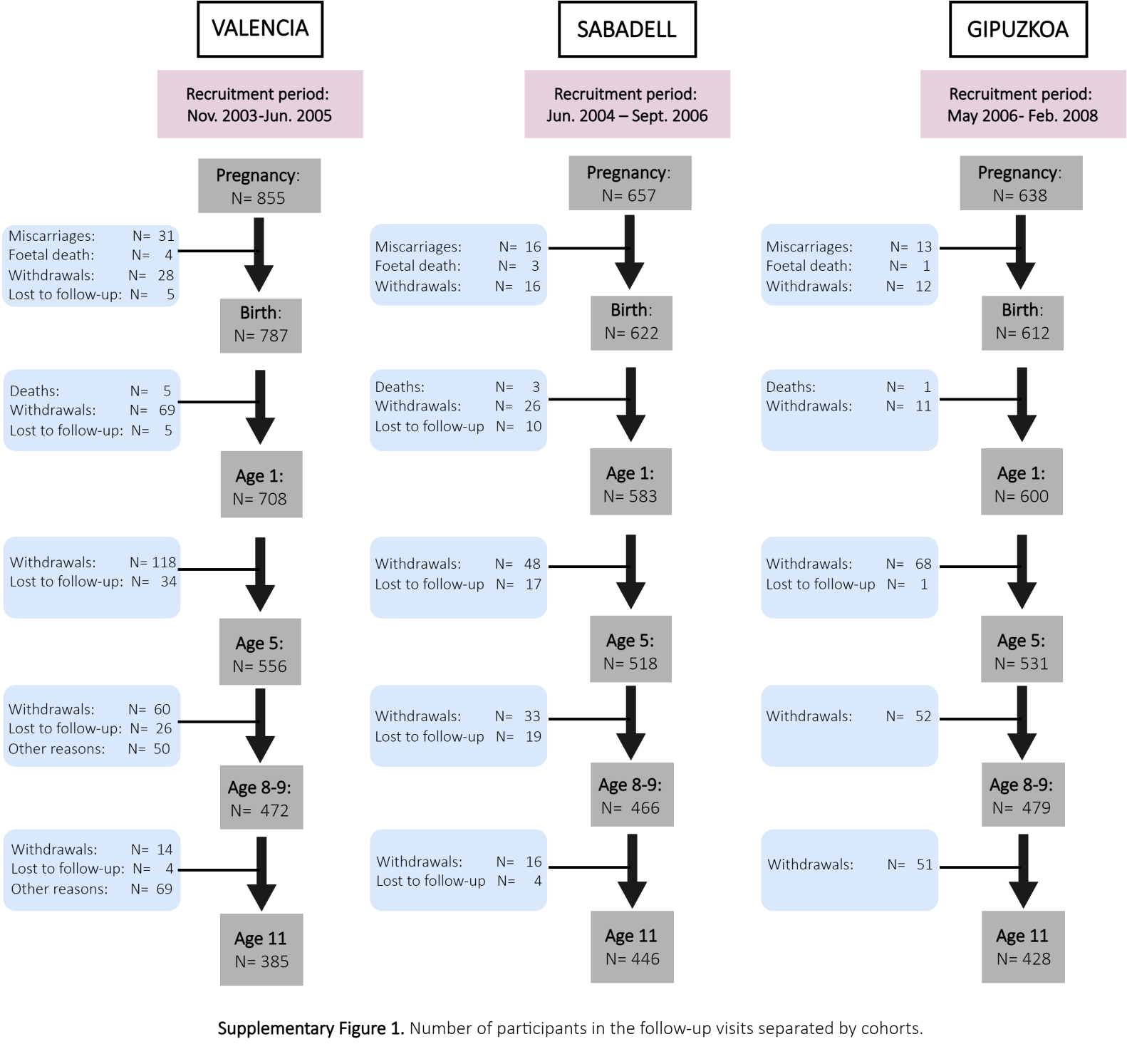


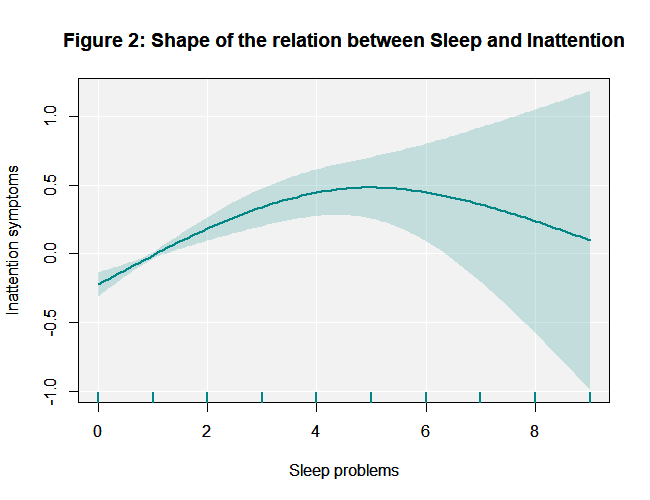

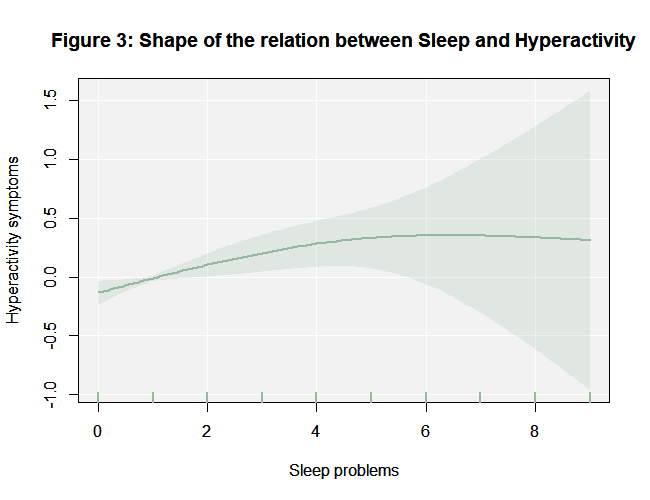

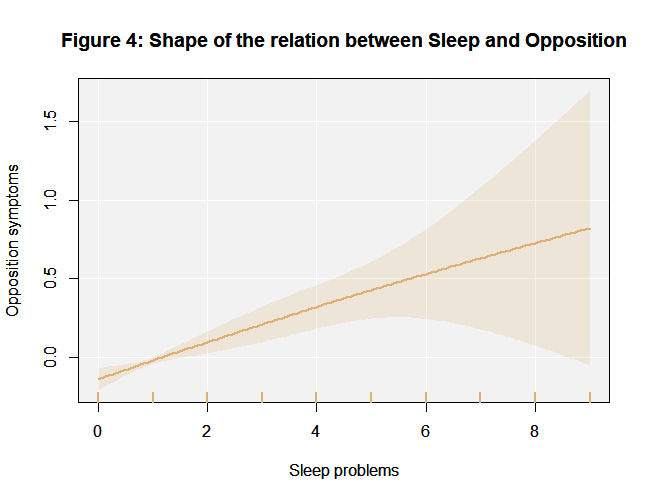

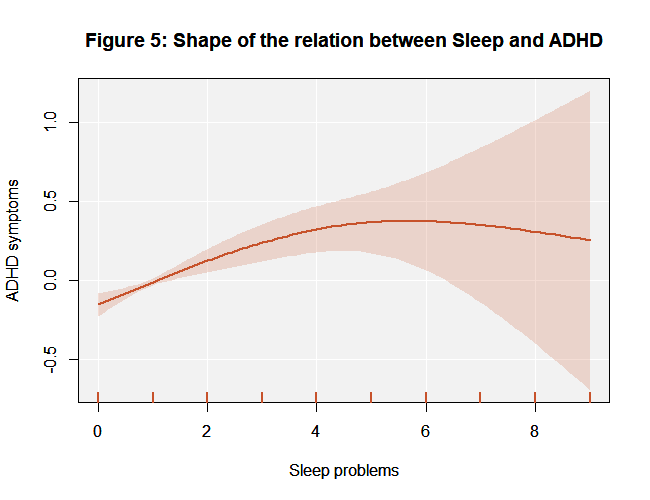


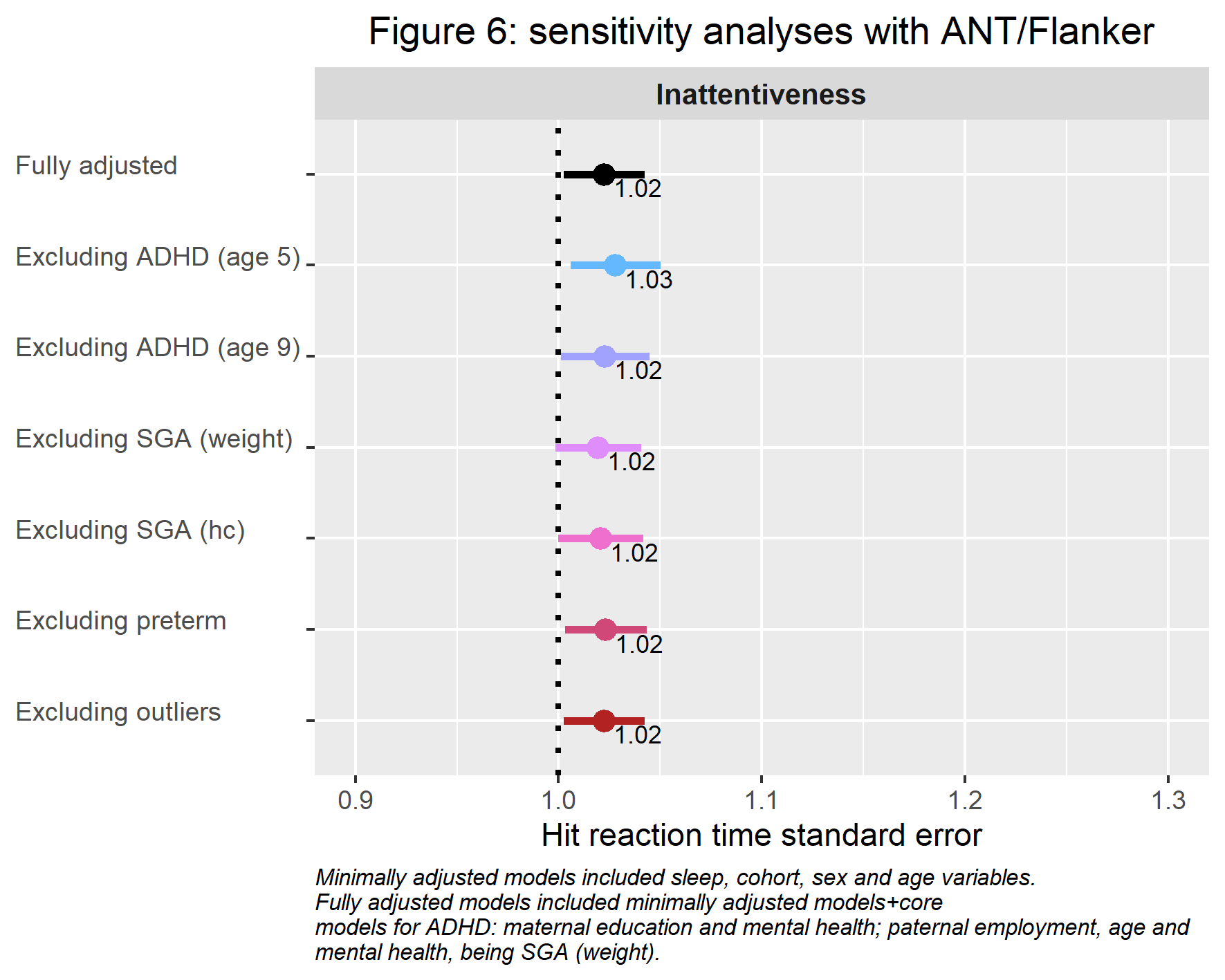


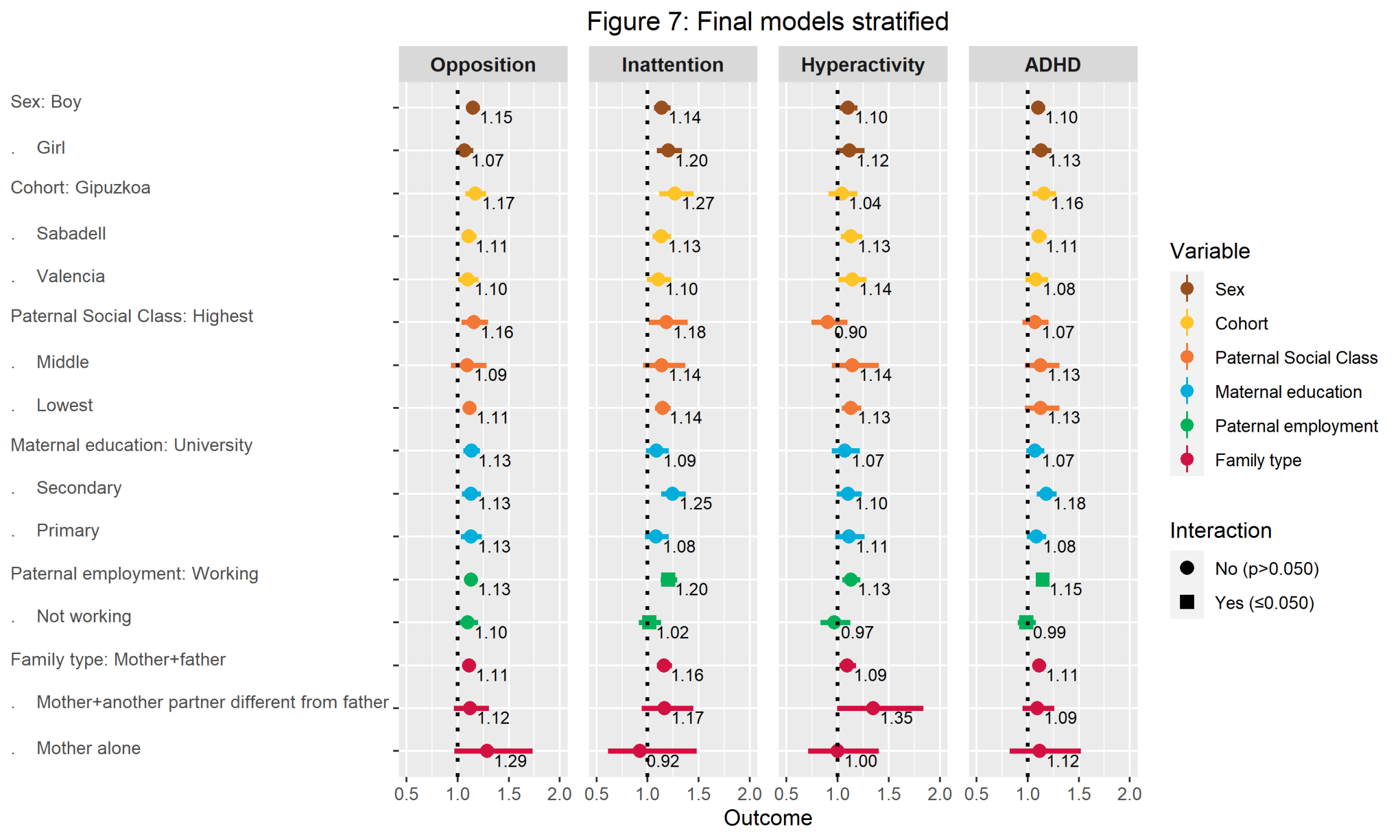

Supplement: Supplementary file 1 — Supplementary file1 (DOCX 12249 KB) [file 431_2023_5145_MOESM1_ESM.docx]
